# Kynurenic Acid Synthesis from D-Kynurenine in the Cerebellum: A Distinct Role of D-Amino Acid Oxidase

**DOI:** 10.3390/cells14131030

**Published:** 2025-07-05

**Authors:** Verónica Pérez de la Cruz, Korrapati V. Sathyasaikumar, Xiao-Dan Wang, Tonali Blanco Ayala, Sarah Beggiato, Dinora F. González Esquivel, Benjamin Pineda, Robert Schwarcz

**Affiliations:** 1 Neurobiochemistry and Behavior Laboratory, National Institute of Neurology and Neurosurgery “Manuel Velasco Suárez”, Mexico City 14269, Mexico; veped@yahoo.com.mx (V.P.d.l.C.); tblanco@innn.edu.mx (T.B.A.); dinora.gonzalez@innn.edu.mx (D.F.G.E.); 2 Maryland Psychiatric Research Center, Department of Psychiatry, University of Maryland School of Medicine, Baltimore, MD 21228, USA; saikumar@som.umaryland.edu (K.V.S.); xiaodanwang1044@163.com (X.-D.W.); 3 Department of Life Sciences and Biotechnology, University of Ferrara, Via Borsari 46, 44121 Ferrara, Italy; sarah.beggiato@unife.it; 4 Neuroimmunology Department, National Institute of Neurology and Neurosurgery “Manuel Velasco Suárez”, Mexico City 14269, Mexico; benjamin.pineda@innn.edu.mx

**Keywords:** D-amino acid oxidase, kynurenic acid, cerebellum, D-kynurenine

## Abstract

The enzymatic formation of kynurenic acid (KYNA), a neuromodulator metabolite of the kynurenine pathway (KP) of tryptophan metabolism, in the mammalian brain is widely attributed to kynurenine aminotransferase II (KATII). However, an alternative biosynthetic route, involving the conversion of D-kynurenine (D-KYN) to KYNA by D-amino acid oxidase (D-AAO), may play a role as well. In the present study, we first confirmed that purified D-AAO efficiently converted D-KYN—but not L-KYN—to KYNA. We then examined KYNA formation from D-KYN (100 µM) in vitro, using tissue homogenates from several human brain regions. KYNA was generated in all areas, with D-AAO-specific production being most effective by far in the cerebellum. Next tested in homogenates from rat cerebellum, KYNA neosynthesis was significantly reduced by D-AAO inhibition, whereas KATII inhibition had no effect. Finally, KYNA production was assessed by in vivo microdialysis in rat cerebellum. Local D-KYN perfusion, alone and in combination with inhibitors of D-AAO (kojic acid) or aminotransferases (AOAA), caused a substantive increase in extracellular KYNA levels. This effect was attenuated dose-dependently by micromolar concentrations of kojic acid, whereas co-perfusion of AOAA (1 mM) was ineffective. Together, our findings indicate that D-AAO should be considered a major contributor to KYNA production in the cerebellum, highlighting region-specific qualitative differences in cerebral KYNA metabolism.

## 1. Introduction

Kynurenic acid (KYNA), an astrocyte-derived metabolite of the kynurenine pathway (KP) of tryptophan metabolism, is increasingly considered to be a versatile and biologically relevant endogenous neuromodulator [1]. KYNA competitively inhibits the glycine co-agonist (glycineB) site of the NMDA receptor [2,3] and, non-competitively, the α7-nicotinic acetylcholine receptor (α7-nAChR) [4] at nanomolar to low micromolar concentrations. At higher, non-physiological concentrations, KYNA antagonizes all ionotropic glutamate receptors [5] and, as shown recently, also targets additional receptors [6,7]. Notably, in vivo fluctuations of endogenous KYNA affect the extracellular concentrations of the major neurotransmitters dopamine [8,9], glutamate [10,11], acetylcholine [12], and GABA [13] in rodent brain. In several cases, these effects have been shown to be initiated by KYNA’s antagonism of α7-nAChRs, which are critically involved in a multitude of brain functions and also participate causally in various neuropathological situations [14,15,16]. In addition and related, brain KYNA plays a significant role in neuroinflammatory processes, which promote tryptophan degradation through the KP and, consequently, stimulate KYNA production [17]. More generally, both increases and decreases in brain KYNA levels cause brain dysfunctions, including excessive neuronal vulnerability (due to KYNA reduction) [18] and a disruption of cognitive processes (due to elevated KYNA) [19,20,21,22]. Of particular interest, and in line with the increasingly appreciated role of KYNA in learning and memory, its down-regulation by selective genetic or pharmacological interventions was shown to improve cognitive performance in experimental animals [23,24,25].

KYNA formation in mammals has been extensively investigated for decades [5,26] and is commonly reported to be catalyzed by kynurenine aminotransferases (KATs), which produce the metabolite irreversibly from its immediate bioprecursor, kynurenine. Of the four KATs identified so far [27,28], KATII (α-aminoadipate transaminase) is primarily responsible for the neosynthesis of rapidly mobilizable KYNA in the brain under physiological conditions [15]. Of note, while KATs preferentially use L-kynurenine (L-KYN) as a substrate, they are not entirely stereospecific and can also convert the D-enantiomer of kynurenine (D-KYN) to KYNA in both peripheral organs and the brain [29,30,31].

Recently, alternative pathways for KYNA biosynthesis have been identified [32,33]. One of these involves the enzymatic conversion of D-KYN by D-amino acid oxidase (D-AAO) [34,35,36,37]. As D-KYN, probably originating from bacterial D-tryptophan [38], is readily detectable in mammals [31], this biosynthetic route clearly deserves careful examination [39].

D-AAO is a peroxisomal enzyme which oxidizes D-amino acids non-specifically through a reaction involving the reduction of its prosthetic group, flavin adenine dinucleotide (FAD), thus producing the corresponding imino acid, which is then hydrolyzed to ammonia and the corresponding α-keto acid [40,41,42,43]. D-amino acids, derived from dietary sources and gut microbiota, are now understood to play substantive roles in modulating neuronal signaling and to participate in cognitive processes [44,45]. Of relevance with regard to KP metabolism, D-AAO not only converts D-tryptophan to L-tryptophan [46] but can also produce D-KYN from D-tryptophan and KYNA from D-KYN [34,39,47]. Interestingly, D-AAO expression and activity in mammalian brain is notably higher in the cerebellum than in the forebrain [48,49]. Furthermore, D-AAO dysregulation has been linked to amyotrophic lateral sclerosis and schizophrenia, i.e., neurological and psychiatric disorders that are associated with altered brain KYNA levels [43,50,51,52]. In light of the apparent translational relevance of KYNA, the present study was designed to determine the respective roles of D-AAO and KATII in the neosynthesis of KYNA from D-KYN in human and rat brain in greater detail.

## 2. Materials and Methods

### 2.1. Materials

KYNA, D-KYN, L-KYN, aminooxyacetic acid (AOAA), kojic acid, and pure porcine kidney D-AAO were purchased from Sigma (St. Louis, MO, USA). All other chemicals were obtained from various commercial suppliers and were of the highest available purity.

### 2.2. Human Brain Tissue

Human brain tissue was obtained from the Maryland Brain Collection, a repository of postmortem tissue maintained in cooperation with the Office of the Chief Medical Examiner of the State of Maryland and housed at the Maryland Psychiatric Research Center. Brain donors (post-mortem interval of ≤22 h) were normal control subjects (age 41 ± 2 years) who were free of neurological or psychiatric disorders. Brain regions were dissected out, and the tissue was stored at −80 °C prior to analysis.

### 2.3. Animals

Male Wistar rats (250–300 g; from the vivarium of National Institute of Neurology and Neurosurgery) were housed in groups of five in acrylic cages with ad libitum access to standard rodent diet and water. They were maintained under controlled conditions of temperature (25 ± 3 °C), humidity (50 ± 10%), and a 12-h light/dark cycle. All experimental procedures followed the guidelines of the Guide for the Care and Use of Laboratory Animals (CICUAL-INNN), as well as the ethical standards of the Ministry of Health of Mexico.

### 2.4. In Vitro Studies

#### 2.4.1. KYNA Production from Pure D-AAO

To assess KYNA production by D-AAO, 1 mg of the pure enzyme was dissolved in 10 mL of 200 mM Tris-HCl buffer, pH 8.4, and 5 µL of the solution were added to 200 µL of the same buffer containing D-KYN or L-KYN (100 µM each) and 0.1 mM flavin adenine dinucleotide (FAD). Where indicated, the reaction mixture also contained the non-specific inhibitor aminotransferase inhibitor AOAA or the non-specific D-AAO inhibitor kojic acid (final concentrations: 1 mM) [53,54]. Samples were then incubated at 37 °C for 2 h, and the reaction was terminated by the addition of 20 µL of 50% trichloroacetic acid and 1 mL of 0.1 N HCl. After centrifugation (14,000× *g*, 10 min), 20 µL of the resulting supernatant were applied to a 3 μm C18 reverse phase high performance liquid chromatography column (80 mm × 4.6 mm; ESA, Chelmsford, MA, USA) using a mobile phase containing 250 mM zinc acetate, 50 mM sodium acetate, and 2% acetonitrile (pH adjusted to 6.2 with glacial acetic acid) and a flow rate of 1.0 mL/min. In the eluate, KYNA was quantitated fluorimetrically (excitation: 344 nm, emission: 398 nm; Perkin Elmer Series 200a fluorescence detector; Perkin Elmer, Waltham, MA, USA). The retention time of KYNA was ~7 min.

#### 2.4.2. KYNA Production from D-KYN in Tissue Homogenates from Human and Rat Brain

In tissue homogenates from human brain (prefrontal cortex, hippocampus, thalamus, striatum, and cerebellum) and from rat cerebellum, the production of KYNA from D-KYN was examined using established assay procedures for measuring KATII and D-AAO activity, respectively. Where indicated, the enzyme inhibitors AOAA or kojic acid (final concentrations: 1 mM) were added to the reaction mixture. In all cases, newly produced KYNA was measured by high-performance liquid chromatography, as described above.

To determine KATII activity, frozen tissues were thawed and homogenized (1:10; *w*/*v*) by sonication in 5 mM Tris-acetate buffer, pH 8.0, containing 10 mM 2-mercaptoethanol and 50 µM pyridoxal-5-phosphate. Eighty micro-liters of the homogenate were then incubated with 100 µM D-KYN for 2 h at 37 °C in 150 mM Tris-acetate buffer, pH 7.4, containing 1 mM pyruvate and 80 µM pyridoxal-5′-phosphate for a total volume of 200 µL [29].

To measure D-AAO activity, thawed tissues were homogenized (1:10; *w*/*v*) by sonication in 200 mM Tris-HCl buffer, pH 8.4, and 80 µL of the homogenate was incubated in the presence of 100 µM D-KYN for 2 h at 37 °C in 200 mM Tris- HCl buffer in a total volume of 200 µL [31].

### 2.5. In Vivo Microdialysis

Rats were assigned to the following experimental groups: (a) D-KYN, (b) D-KYN + AOAA, and (c) D-KYN + kojic acid. Each group consisted of five to six animals. Group allocation was randomized using a simple randomization method. Rats were anesthetized with ketamine (80 mg/kg, i.p.) and xylazine (10 mg/k, i.p.) and placed in a stereotaxic frame. A guide cannula (MAB 2.14.G, SciPro Inc., Sanborn, NY, USA) was positioned in the cerebellum (AP: 10.6 mm anterior to bregma, L: 1.6 mm from the midline, V: 1.2 mm below the dura) and secured to the skull with stainless steel screws and acrylic dental cement. After surgery, the animals were allowed to recover and were housed individually in acrylic cages with full access to food and water. The next day, a microdialysis probe (MAB 9.14.2, membrane length: 2 mm; SciPro) was inserted and connected to a microperfusion pump set at a flow rate of 1.5 μL/min. The freely moving animals were perfused with Ringer solution (pH 6.7) containing 144 mM NaCl, 4.8 mM KCl, 1.2 MgSO_4_, and 1.7 mM CaCl_2_ for 2.5 h to establish a baseline. D-KYN (100 µM) was then perfused locally for 2 h alone or combined with kojic acid (1 mM) or AOAA (1 mM). Subsequently, perfusion with Ringer solution continued for 4 h. Microdialysis samples were collected every 30 min. Ten micro-liters of the microdialysate were applied to a 5 μm C18 reverse-phase column (Shimadzu Nexcol C18 5 µm, 50 mm × 3.0 mm, Shimadzu, Columbia, MD, USA) using a mobile phase containing 250 mM zinc acetate, 50 mM sodium acetate, and 2% acetonitrile (pH adjusted to 6.2 with glacial acetic acid), with a flow rate of 0.5 mL/min. KYNA was quantitated fluorometrically (excitation: 344 nm, emission: 398 nm; RF-20Axs Shimadzu fluorescence detector; Shimadzu, Tokyo, Japan). The retention of KYNA was ~8 min. Data were not corrected for recovery rate from the microdialysis probe and are presented as a percent increase from the baseline.

### 2.6. Statistical Analysis

Data are expressed as the mean ± standard error of the mean (SEM). Comparisons between groups used the one-way ANOVA followed by Bonferroni’s post-hoc test. Two-way ANOVA with Bonferroni’s multiple comparisons test for each timepoint between treatments was used for in vivo microdialysis experiments. Significance was set at *p* < 0.05, using GraphPad Prism 10 version 10.5.0 (GraphPad, San Diego, CA, USA).

## 3. Results

### 3.1. Pure D-AAO Produces KYNA from D-KYN

**Figure 1:**
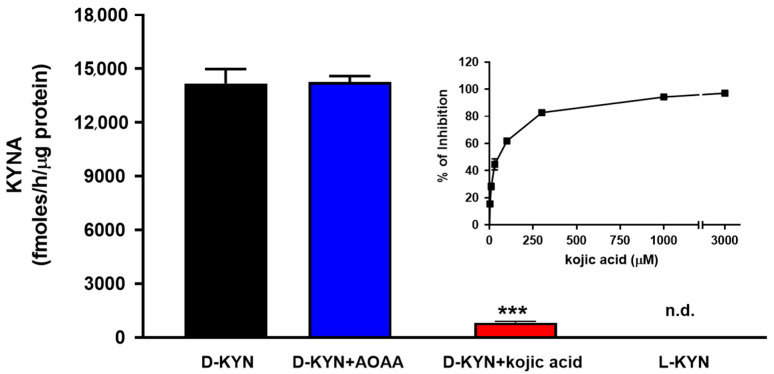
Pure D-AAO produced KYNA from D-KYN in vitro. D-AAO was incubated with 100 µM D-KYN or L-KYN for 2 h at 37 °C in the presence or absence of AOAA (1 mM) or kojic acid (1 mM). Data are the mean ± SEM of three experiments. *** *p* < 0.001 vs. D-KYN alone (one-way ANOVA followed by Bonferroni’s post-hoc test). n.d.: not detectable. Inset: kojic acid dose-dependently inhibited the neosynthesis of KYNA from D-KYN.

Incubation of D-KYN with pure D-AAO led to significant KYNA production, and the neosynthesis of KYNA was dose-dependently reduced by the addition of the D-AAO inhibitor, i.e., kojic acid (~95% inhibition at 1 mM). In contrast, co-incubation with the aminotransferase inhibitor, i.e., AOAA (1 mM), had no effect on KYNA production, and no KYNA formation was observed after incubation with L-KYN (Figure 1).

### 3.2. KYNA Production from D-KYN in Human Brain Tissue Homogenates In Vitro

**Figure 2:**
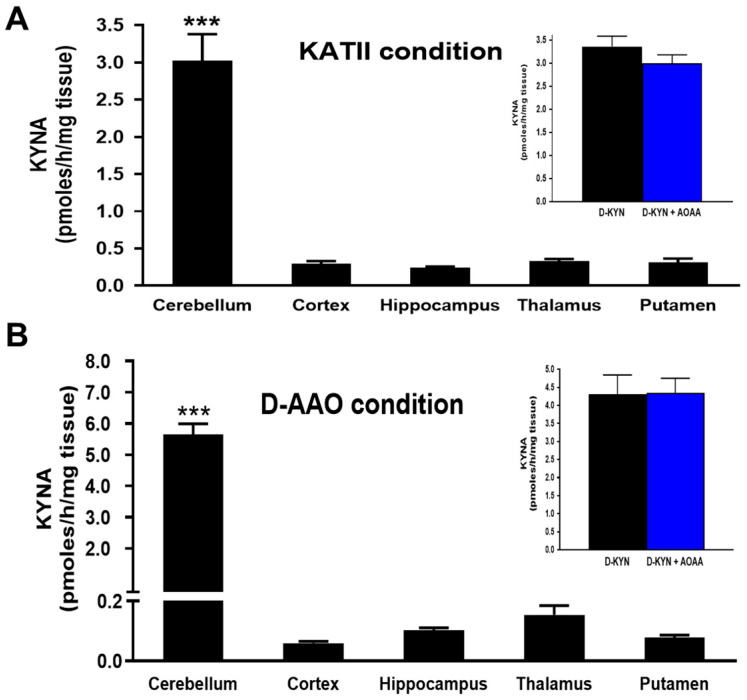
KYNA neosynthesis from D-KYN across several human brain regions under optimal experimental conditions for measuring KATII (**A**) and D-AAO (**B**) activity, respectively. Tissue homogenates were incubated with 100 µM D-KYN for 2 h at 37 °C. See text for additional details. Inserts: the aminotransferase inhibitor AOAA (1 mM) did not affect the neosynthesis of KYNA in the cerebellum under either of the two assay conditions. Data are the mean ± SEM (*n* = 5). *** *p* < 0.001 vs. all other brain regions (one-way ANOVA followed by Bonferroni’s post-hoc test).

Next, we studied the de novo production of KYNA from D-KYN in vitro in tissue homogenates obtained from five different human brain regions (cortex, hippocampus, thalamus, putamen, and cerebellum). To this end, samples were incubated with D-KYN under two experimental conditions, favoring KATII activity and D-AAO activity, respectively. KYNA formation was detected in all brain areas in both cases, with KATII conditions being approximately half as effective (Figure 2). Notably, the cerebellum showed more than 10-fold greater KYNA production than the other brain areas under both incubation conditions. The addition of 1 mM AOAA did not reduce cerebellar KYNA synthesis (Figure 2, insets).

### 3.3. Roles of D-AAO and KATII in the Production of KYNA from D-KYN in the Rat Cerebellum In Vitro

**Figure 3:**
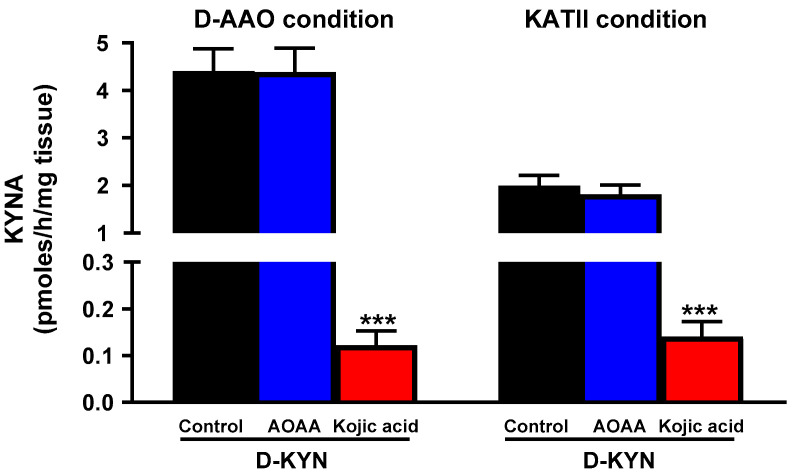
In vitro production of KYNA from D-KYN in rat cerebellar homogenates under optimal conditions favoring D-AAO and KATII activity, respectively: effect of AOAA and kojic acid. Tissue homogenates were incubated with 100 µM D-KYN for 2 h at 37 °C under D-AAO or KATII condition in the presence or absence of AOAA (1 mM) or kojic acid (1 mM). See text for additional details. Data are the mean ± SEM (*n* = 5–6). *** *p* < 0.001 vs. D-KYN alone (one-way ANOVA followed by Bonferroni’s post-hoc test).

The remarkably greater efficacy of the conversion of D-KYN to KYNA in the human cerebellum prompted us to study the relative contributions of D-AAO and KATII in tissue homogenates from the rat cerebellum. These experiments revealed a pattern similar to that seen in the human cerebellum (cf. above), with approximately double the efficacy under conditions favoring D-AAO activity (Figure 3). Here, too, incubation with AOAA did not affect KYNA levels, whereas the D-AAO inhibitor kojic acid essentially abolished KYNA production.

### 3.4. Roles of D-AAO and KATII in the Production of KYNA from D-KYN in Rat Cerebellum In Vivo

**Figure 4:**
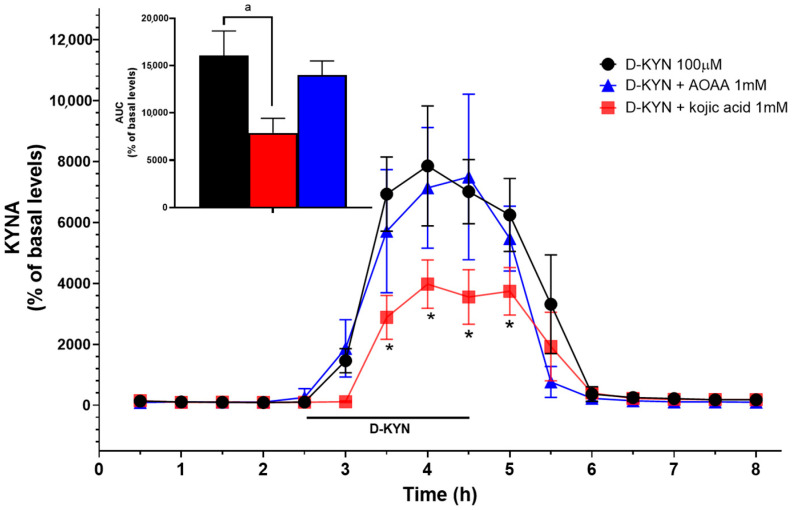
Effect of D-KYN on extracellular KYNA levels in the rat cerebellum in vivo. D-KYN (100 µM) was applied by reverse dialysis (bar), alone or together with AOAA and kojic acid, and KYNA was measured in microdialysate samples. See text for additional details. Data are the mean ± SEM (*n* = 5 per group). Inset: Areas under the curve (AUC) were calculated from 2 h to 8 h for each rat. Data are the mean ± SEM (*n* = 5–6 per group). ^a^ *p* < 0.05 vs. baseline (one-way ANOVA followed by Bonferroni’s post-hoc test), * *p* < 0.05 vs. D-KYN alone (two-way ANOVA followed by Bonferroni’s post-hoc test).

Finally, we investigated the impact of D-KYN on extracellular KYNA concentrations in rat cerebellum by in vivo microdialysis. To this end, we applied 100 µM of D-KYN locally by reverse dialysis. As shown in Figure 4, D-KYN infusion caused a substantive increase in extracellular KYNA (basal levels: 3.1 ± 1.9 nM), reaching a maximum (237.6 ± 19.1 nM) after approximately 1.5 h. In separate animals, D-KYN was co-perfused with either 1 mM AOAA or 1 mM kojic acid. Co-application of AOAA had no noticeable effect on KYNA levels, whereas kojic acid significantly inhibited the increase in KYNA (128.1 ± 17.8 nM) formation, reducing the area under the curve by approximately 50% (Figure 4, inset).

## 4. Discussion

The present study provides new insights into the enzymatic pathways involved in KYNA production from D-KYN, particularly highlighting the significant role of D-AAO in the cerebellum in this context. Our findings confirm that KYNA can be produced from D-KYN both in vitro and in vivo, with a clear preference for D-AAO- vs. KATII-mediated conversion. The human cerebellum was identified as a key brain region for D-AAO-catalyzed KYNA synthesis, showing considerably higher production than the cortex, hippocampus, thalamus, and putamen. Given the role of D-amino acids in the regulation of neuronal signaling, cognition, and aging [55,56], the identification of D-KYN as a crucial precursor of KYNA in the cerebellum suggests new avenues for therapeutic strategies in disorders associated with altered D-AAO activity and/or KYNA dysregulation.

Our experiments with purified D-AAO confirmed the ability of the enzyme to effectively transform D-KYN to KYNA, corroborating previous reports that D-AAO catalyzes the transamination of D-KYN and that L-KYN is not a viable substrate [31,47]. The enzymatic mechanism appears to involve deamination of D-KYN followed by spontaneous ring closure to form KYNA [30,57,58]. A key novel observation in the present study was not only the disproportionally high effect seen in the cerebellum, which parallelled the high expression and activity of D-AAO in this region [48,59,60,61], but also the demonstration that D-KYN-induced KYNA synthesis in the cerebellum was not generated by KATs, since the co-administration of AOAA had no effect either in vitro or in vivo. This was in marked contrast to the striatum, where microdialysis experiments in rats showed that the conversion of D-KYN to KYNA was partially mediated by enzymatic transamination, since co-infusion with AOAA resulted in a 40% inhibition of the process [29]. These region-dependent differences in KYNA production can be explained by the distinct distribution of the enzymes involved: in the cerebellum, D-AAO expression is significantly higher than that of KATII, making D-AAO the dominant contributor to KYNA synthesis from D-KYN. Conversely, in the striatum, KATII is highly expressed and thus plays a major role in KYNA formation from D-KYN. These findings highlight the importance of regional enzymatic profiles, which could become particularly relevant under pathological conditions, i.e., where alterations in D-AAO or KATII expression or activity may exacerbate or otherwise modulate region-specific changes in KYNA levels and, consequently, neuronal function.

Although D-amino acids are generally present in low abundance in mammalian tissues [62], their levels and metabolism can change substantially during dysbiosis or infections, as many microorganisms possess the enzymatic machinery to produce and process D-amino acids [44,63]. In these conditions, the availability of D-KYN may increase, potentially enhancing KYNA production via D-AAO which is highly expressed in the cerebellum, with possible consequences for cerebral function [64]. Notably, the cerebellum is one of the brain regions with the lowest endogenous levels of KYNA under physiological conditions [65].

The reciprocal relationship of the cerebellum with brain regions involved in cognition, executive function, and emotion regulations (i.e., the cortex, thalamus, hippocampus, and parietal association areas) is well established [66,67]. Although the role of cerebellar KYNA in brain (dys) function is still largely unexplored, its biological relevance clearly deserves detailed future evaluation. Specifically, in view of the cerebellum functional connectivity with the prefrontal cortex [68,69], irregular cerebellar KYNA synthesis may contribute to the dysregulation of dopaminergic and glutamatergic signaling in cortical areas, leading to cognitive impairments and behavioral abnormalities. Of note in this context, elevated levels of KYNA are found in the forebrain and cerebrospinal fluid of patients with schizophrenia [70,71] and Alzheimer’s disease (see [72] for review), i.e., major brain disorders that are associated with cognitive impairments. Moreover, high KYNA concentrations may play a role in cognitive impairments seen during the aging process [73,74].

Future studies therefore ought to focus on the intricacies of KYNA biology in the cerebellum under physiological and particularly under pathological conditions, considering developmental patterns as well as possible species and sex differences. Experimental outcome measures should include detailed anatomical and electrophysiological analyses to clarify the cellular localization of KATII and D-AAO (both of which have been reported to be present in cerebellar astrocytes [59,75]) and the functional interactions of these cells with α7-nACh, NMDA, and, possibly, additional receptors (see Section 1). Based on the present results, showing essentially complete separation of KYNA neosynthesis from L-KYN and D-KYN by KATII and D-AAO, respectively, these studies can be expected to provide informative insights into the roles of the two kynurenine enantiomers in the cerebellum and beyond.

Pharmacological modulation of brain KYNA levels has been repeatedly proposed as a therapeutic strategy in a number of neuropsychiatric diseases, in most cases targeting KATII and other enzymes of the classic KP of tryptophan degradation (see [15] for review). With particular emphasis on regulating the enzyme in the cerebellum, and also in view of the fact that its expression and function is abnormal in the brain of persons with schizophrenia [49,76], the present study suggests that attention should now also be paid to D-AAO in this respect. Notably, D-AAO manipulations aimed at clinical use have so far focused almost exclusively on controlling the levels of its substrate D-serine, a critical glycineB receptor agonist [77]. Also from a translational perspective, future studies will therefore need to consider the respective effects of pharmacologically induced fluctuations in D-KYN and D-serine levels and function in physiological and pathological settings.

## 5. Conclusions

The present study revealed substantive region-specific differences in KYNA neosynthesis from D-KYN in mammalian brain, demonstrating a prominent role of D-AAO in KYNA production in the cerebellum. In light of the increasingly recognized role of D-AAO in brain physiology and pathology, our findings open heretofore unappreciated opportunities for the development of targeted therapies of brain disorders involving abnormal KYNA function.

## Data Availability

Data will be made available upon reasonable requests to the corresponding author.
